# Research—What is it good for? Absolutely nothing… unless it is used to inform policy and practice

**DOI:** 10.1002/cl2.1276

**Published:** 2022-09-13

**Authors:** Howard White, Vivian Welch

Recent strategies of the Campbell Collaboration have stressed not just increasing the production of high‐quality reviews, but also increasing the use of those reviews by decisionmakers. However, the things that researchers usually do—publishing in journals and speaking at academic conferences—will not reach decisionmakers. A short plain language summary is embedded in every Campbell review, but this is still of little or no use if those summaries remain on our website, undiscovered, rather than in the hands of decisionmakers.

Campbell has been working to increase the policy uptake of evidence from Campbell reviews by building on two recent trends. First, there is increasing recognition of the need to involve stakeholders, especially decisionmakers, at the early stages of any research. The second trend is the rise of knowledge brokering or knowledge translation—that is, the need to take the research one step further in ‘translating’ the research findings into forms which are discoverable, understandable and usable by decisionmakers.

The main way in which Campbell is helping to build policy uptake is to work with what we call user commissioners. These are commissioners of research who are not traditional research funders, funding research for research's sake, but organisations who have a demand for knowledge to inform their own work. These user commissioners include the What Works movement, whose members are responsible for developing evidence‐based decisionmaking products. They work with their stakeholders in diverse sectors, such as youth employment, youth offending, homelessness and education. They also work with other user commissioners in the World Health Organization, development banks and governmental departments who inform national implementation strategies.

Campbell's approach includes the strategic use of evidence and gap maps, to build the evidence architecture (White, 2021). In this approach, we first undertake an evidence and gap map of the sector of interest with the user commissioner and with the engagement of other stakeholders such as citizens, practitioners and decisionmakers. For example, we developed the maps of effectiveness and implementation evidence for people who are homeless or vulnerably housed in collaboration with the Centre for Homelessness Impact in the UK, as well as other key stakeholders (White et al., 2020). Another example is the evidence and gap map on home‐based health and social care, developed in collaboration with the World Health Organization, practitioners and citizens (Welch et al., 2021).

These maps may serve several purposes. If the evidence base is strong, there may be existing high‐quality systematic reviews which can be used to produce evidence‐based decisionmaking tools. The first edition of the Youth Endowment Fund (YEF) for England and Wales – youth offending toolkit (https://youthendowmentfund.org.uk/toolkit-about/) includes ratings of cost, evidence quality and impact on violent crime. This toolkit contained 17 strands, with each strand based on an existing systematic review, many of which are Campbell reviews. To build the toolkit further, we are now commissioning Campbell systematic reviews on behalf of YEF in areas where there are many studies but no systematic reviews. This is also happening with the Centre for Homelessness Impact: we found very few systematic reviews, so the first step was to commission some (via Campbell UK & Ireland, based at Queen's University Belfast), such as the review on accommodation‐based interventions (Keenan et al., 2021).

Other organisations have asked us to advise: one example is the World Health Organization's work on elder abuse and strategies to mitigate social isolation and loneliness for older adults (Welch et al., 2022).

User commissioners, such as the UK What Works Network, have credibility and significant connections amongst stakeholder communities, which helps ensure use of these evidence products based on Campbell reviews and other work by Campbell. For example, after only 1 year, the YEF toolkit has high recognition amongst target stakeholders. Youth Offending Teams and local authorities use it to inform the design of local strategies to tackle youth offending. Several new government funds have tied resource allocation to interventions proven to work by the evidence in the YEF toolkit. And the UK Home Office has said that 20% of spending by Violence Reduction Units must be for interventions shown to be effective in the in the YEF toolkit. These are all cases of research evidence from systematic reviews driving decisions on the ground, improving lives on the front line.

We are now exploring how to accelerate inclusion of Campbell reviews in practical guidelines in the social sectors, using methods such as Grading of Recommendations Assessment, Development and Evaluation (GRADE). Our user commissioner partners are asking about guidance for practitioners, but Campbell reviews do not do this, since they are intended as unbiased syntheses of the evidence. Guidance requires explicit and transparent judgement of additional factors such as values, acceptability, feasibility and costs. So we are working with organisations including the UK National Institute for Health and Care Excellence (NICE), the World Health Organization and the Public Health Agency of Canada to identify best practice and challenges of rigorous guideline development in the social sectors. We held an exploratory meeting in January 2021, and there will be a special session at the upcoming Guidelines International Network meeting (21–24 September 2022), https://g-i-n.net/conference_2022/welcome/.

Campbell's vision is ‘better evidence for a better world’. Engaging with user commissioners allows us to achieve that. Let's make the world a better place ‘one systematic review at a time’ (Stewart, 2014).
